# Systematic review and metaanalysis of genetic association studies of urinary symptoms and prolapse in women

**DOI:** 10.1016/j.ajog.2014.08.005

**Published:** 2015-02

**Authors:** Rufus Cartwright, Anna C. Kirby, Kari A.O. Tikkinen, Altaf Mangera, Gans Thiagamoorthy, Prabhakar Rajan, Jori Pesonen, Chris Ambrose, Juan Gonzalez-Maffe, Phillip Bennett, Tom Palmer, Andrew Walley, Marjo-Riitta Järvelin, Chris Chapple, Vik Khullar

**Affiliations:** a Department of Epidemiology and Biostatistics, Imperial College London, London, England, United Kingdom; b Department of Urogynecology, Imperial College London, London, England, United Kingdom; c Department of Genomics of Common Disease and Molecular Genetics and Genomics, Imperial College London, London, England, United Kingdom; d Clinical Trials Unit, Imperial College London, London, England, United Kingdom; e Institute for Reproductive and Developmental Biology, Imperial College London, London, England, United Kingdom; f Department of Urogynecology, King’s College London, London, England, United Kingdom; g University College London Medical School, London, England, United Kingdom; h Department of Urology Research, University of Sheffield, Sheffield, England, United Kingdom; i Division of Health Sciences, Warwick Medical School, University of Warwick, Coventry, England, United Kingdom; j Beatson Institute for Cancer Research, University of Glasgow, Glasgow, Scotland, United Kingdom; k Department of Reproductive Medicine, University of California, San Diego, School of Medicine, and Department of Obstetrics and Gynecology, Kaiser Permanente, San Diego, CA; l Departments of Urology and Public Health, Helsinki University Central Hospital and University of Helsinki, Helsinki, Finland; m Department of Urology, University of Tampere, Tampere, Finland; n Institute of Health Sciences and Biocenter Oulu, University of Oulu, Finland; o Department of Clinical Epidemiology and Biostatistics, McMaster University, Hamilton, Ontario, Canada

**Keywords:** genetics, incontinence, lower urinary tract symptoms, overactive bladder, prolapse, systematic review

## Abstract

**Objective:**

Family studies and twin studies demonstrate that lower urinary tract symptoms and pelvic organ prolapse are heritable. This review aimed to identify genetic polymorphisms tested for an association with lower urinary tract symptoms or prolapse, and to assess the strength, consistency, and risk of bias among reported associations.

**Study Design:**

PubMed and HuGE Navigator were searched up to May 1, 2014, using a combination of genetic and phenotype key words, including “nocturia,” “incontinence,” “overactive bladder,” “prolapse,” and “enuresis.” Major genetics, urology, and gynecology conference abstracts were searched from 2005 through 2013. We screened 889 abstracts, and retrieved 78 full texts. In all, 27 published and 7 unpublished studies provided data on polymorphisms in or near 32 different genes. Fixed and random effects metaanalyses were conducted using codominant models of inheritance. We assessed the credibility of pooled associations using the interim Venice criteria.

**Results:**

In pooled analysis, the rs4994 polymorphism of the *ADRB3* gene was associated with overactive bladder (odds ratio [OR], 2.5; 95% confidence interval [CI], 1.7–3.6; n = 419). The rs1800012 polymorphism of the *COL1A1* gene was associated with prolapse (OR, 1.3; 95% CI, 1.0–1.7; n = 838) and stress urinary incontinence (OR, 2.1; 95% CI, 1.4–3.2; n = 190). Other metaanalyses, including those for polymorphisms of *COL3A1,LAMC1,MMP1,MMP3,* and *MMP9* did not show significant effects. Many studies were at high risk of bias from genotyping error or population stratification.

**Conclusion:**

These metaanalyses provide moderate epidemiological credibility for associations of variation in *ADRB3* with overactive bladder, and variation of *COL1A1* with prolapse. Clinical testing for any of these polymorphisms cannot be recommended based on current evidence.

*Female pelvic floor disorders*, an umbrella term including urinary incontinence, bladder storage symptoms, and pelvic organ prolapse (POP) are highly prevalent.^1,2^ Almost one quarter of adult women report at least one clinically meaningful pelvic floor disorder,^1,3^ with frequent overlap between conditions.^4,5^ These conditions are associated with a range of comorbidities,^6-8^ and have a substantial impact on quality of life.^9-11^ There are strong associations with both age and obesity,^12-15^ and thus the population burden of these conditions will increase with future demographic shifts.

The existence of inherited risk factors for pelvic floor disorders has been recognized for more than 150 years,^16^ and there is clear familial aggregation for these conditions. Having an affected first-degree relative with incontinence or prolapse is associated with an approximately 2- to 3-fold increased risk of developing either condition, with effects measurable for all major subtypes of incontinence, and for anterior, apical, and posterior compartment prolapse.^17-21^ A relevant family history is associated with both earlier onset, and more rapidly progressive symptoms.^22,23^

Family studies provide limited information on heritability, as they do not control for shared exposure to environmental risk factors. Twin studies have been used to formally quantify the heritability of lower urinary tract symptoms (LUTS) or prolapse. In a sample of 16,886 Swedish twins aged >50 years, heritability was estimated as 41% for stress incontinence surgery, and 43% for prolapse surgery.^24^ Similarly for twins aged 20-46 years from the same cohort (n = 4550), heritability was estimated as 34% for stress incontinence, 37% for urgency incontinence, and 48% for nocturia.^25^ Among a cohort of 2336 women enrolled in the Danish Twin Register,^26^ heritability ranged with age from 42-49% for urgency incontinence, 27-55% for mixed incontinence, and up to 39% for stress incontinence.

Identification of the genetic variants underlying the heritability of these conditions would provide useful markers for clinical risk, prognosis, and treatment response. In addition, however, the insights provided should help explain the pathogenesis of these complex diseases, potentially offering new drug targets and preventative strategies. The aim of this systematic review was therefore to assess which candidate polymorphisms and/or candidate genes had been tested for an association with POP or LUTS in women, and to assess the strength, consistency, and potential for bias among published associations.

## Materials and Methods

### Eligibility criteria

The review protocol was prospectively registered (PROSPERO 2011:CRD42012001983).^27^ We prespecified inclusion of both case-control and cross-sectional designs, with both population-based samples and other sampling methods. We included association studies testing for any genetic polymorphism at the nucleotide level, including single-nucleotide polymorphisms (SNPs), deletions, duplications, and copy-number variants, but excluded larger microscopic variants at the karyotype level.

There are no gold standard diagnostic methods for either stress urinary incontinence (SUI) or other LUTS, as these are largely subjective symptomatic diagnoses. For POP, validated staging systems, including POP Quantification, have been widely used, but again there is no universally accepted criterion for diagnosis. We therefore expected to accept diagnostic criteria for LUTS and prolapse as specified within each study. In view of heterogeneity in definitions across studies, we tested for heterogeneity between studies with different criteria in different settings. We accepted definitions based on symptom questionnaires, clinical examination, urodynamics, or other validated assessments. We considered the population of interest as women aged ≥18 years.

### Search strategy

We combined searches from PubMed, HuGE Navigator, and an extensive selection of genetic, urological, and urogynecological conference reports. We searched PubMed up to May 1, 2014, without language restrictions, using a combination of genetic and phenotype key words and Medical Subject Headings (MeSH) terms: *(polymorphism OR SNP OR CNV OR “copy number variation” OR mutation OR genetic OR chromosome OR VNTR OR InDel OR microsatellite) AND (nocturia OR LUTS OR incontinence OR urgency OR “overactive bladder” OR prolapse OR “Lower Urinary Tract Symptoms”[Mesh] OR “Urinary Incontinence”[MeSH] OR “enuresis”[Mesh] OR “Pelvic Organ Prolapse”[MeSH]) NOT mitral NOT carcinoma[Title] NOT cancer[Title] NOT (animals[mh] NOT humans[mh])*.

We searched HuGE Navigator, also through to May 1, 2014, using the following phenotype indexing terms: *(“urination disorders” OR “urinary incontinence” OR “pelvic organ prolapse”)*.

In addition we searched conference abstracts for annual meetings of the American Society of Human Genetics, American Urological Association, American Urogynecologic Society, European Association of Urology, European Society of Human Genetics, International Continence Society, International Urogynecological Association, and Society of Gynecologic Surgeons 2005 through 2013.

### Screening and data extraction

We developed standardized data forms for this study, and conducted pilot screening and data extraction training exercises to achieve a high level of consensus between reviewers. All screening and data extraction was then performed independently and in duplicate by methodologically trained reviewers. Reviewers screened study reports by first screening titles and abstracts to select papers for full-text assessment, then screening full-text papers to confirm eligibility of the articles. Screening discrepancies were resolved by adjudication. We hand searched reference lists of all included articles, applying the same standardized screening process. When >1 report was identified for the same association in the same study population, we included the publication with the largest sample size.

We contacted study authors by email, with a reminder after 1 month, for clarifications, additional information about methodology, and additional subgroup analyses where necessary. Data extracted included information on the setting for each study, details of the sampling strategy and sampled populations (age, parity, ethnic/racial composition, and body mass index), the overall sample size and proportion genotyped, the outcome assessments used and phenotypic definitions, the genotyping method employed, and the genotyping quality control applied. Where possible we extracted or requested from authors full genotype frequencies among both cases and controls.

### Statistical analysis and risk of bias assessments

For polymorphisms assessed in ≥2 studies for the same phenotype assessed with similar case definitions, we conducted fixed or random effects metaanalyses as appropriate using the Metan^28^ package (Stata 12.1; StataCorp, College Station, TX). In all cases, we worked from genotype or allele frequencies, rather than using precalculated effect sizes. We did not pool data from studies with mixed male and female samples, unless results stratified by sex were available. We did not pool data from studies with composite case definitions (ie, any urinary incontinence) with those with simple case definitions (ie, SUI). In the absence of a clear rationale supporting any specific model of inheritance, we used the allelic association test/codominant models of inheritance for all polymorphisms. We assessed the credibility of pooled associations using the interim Venice criteria^29^ (Appendix; Supplementary Figure). We used the I^2^ statistic as a measure of between study heterogeneity. We recalculated the power of each study, and retested for departure from Hardy-Weinberg equilibrium. We made assessments of risk of bias in phenotype definitions, genotyping, and population stratification. We used the Harbord test of funnel plot asymmetry, and the significance chasing bias test^30^ to investigate possible reporting biases. Reporting of this review complies with recommendations both of the HuGE Handbook, and the PRISMA statement.^31,32^

## Results

### Search outcomes

**Figure 1:**
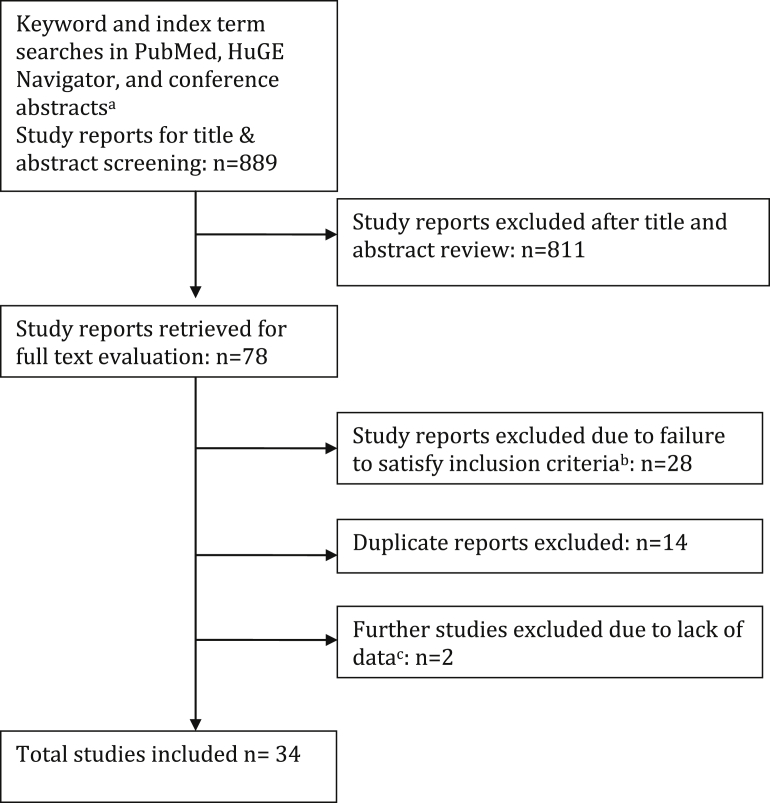
Flowchart outlining literature search and article evaluation process ^a^ American Society of Human Genetics, American Urological Association, American Urogynecologic Society, European Association of Urology, European Society of Human Genetics, International Continence Society, International Urogynecological Association, and Society of Gynecologic Surgeons abstracts 2005 through 2014, using online search interfaces and/or full text search of abstract book PDFs; ^b^ Includes studies enrolling only men (n = 122), enrolling only children (n = 2), narrative reviews or letters (n = 12), inapplicable phenotype (n = 2), and other study designs including pharmacogenetic studies, gene expression studies, or methylation studies (n = 8); ^c^ Authors contacted by email for additional data from 18 studies. *Cartwright. Genetic association studies of LUTS and POP. Am J Obstet Gynecol 2015*.

We screened 889 abstracts, and retrieved 78 full texts (Figure 1). In all, 27 published studies and 7 unpublished studies provided data (Table 1) regarding polymorphisms in or near 32 different genes (Supplementary Table 1). Most research interest has focused on variation in genes implicated in extracellular matrix organization and disassembly, with particular focus on collagen and metalloendopeptidase genes (Supplementary Table 2). A number of studies also addressed a variety of steroid hormone receptor genes. All studies investigated POP, SUI, or overactive bladder, with no available data on other individual LUTS.

Quantitative syntheses were possible for 11 polymorphisms in or near 7 genes: beta 3 adrenoceptor (*ADRB3*); collagen, type I, alpha 1 (*COL1A1*); collagen, type 3, alpha 1 (*COL3A1*); laminin gamma 1 (*LAMC1*); matrix metalloproteinase-1 (*MMP1*); matrix metalloproteinase-3 (*MMP3*); and matrix metalloproteinase-9 (*MMP9*).

### *ADRB3*

**Figure 2:**
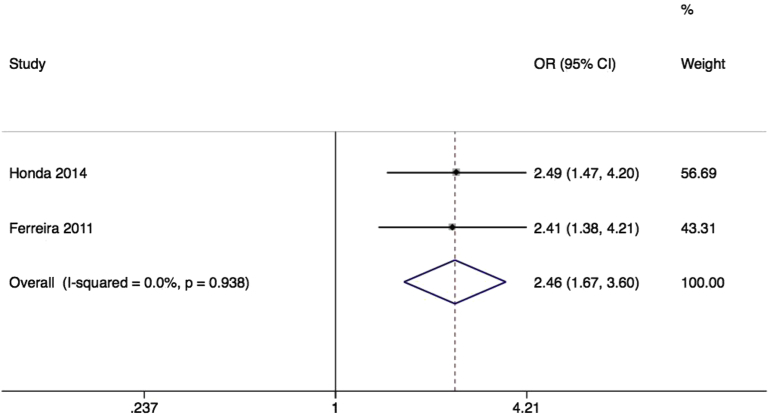
Forest plot of rs4994 SNP of ADRB3 and overactive bladder Forest plot of studies^37,38^ reporting associations between rs4994 single-nucleotide polymorphism (SNP)* of beta 3 adrenoceptor gene and overactive bladder. *RefSNP alleles C/T. Plot presented as risk associated with minor allele C. *CI*, confidence interval; *OR*, odds ratio. *Cartwright. Genetic association studies of LUTS and POP. Am J Obstet Gynecol 2015*.

Variation in the beta-3 adrenoceptor, particularly of the rs4994 SNP, also known as Trp64Arg, has been extensively investigated in association with obesity, type 2 diabetes mellitus, and other metabolic syndrome phenotypes. The beta-3 adrenoceptor is highly expressed in bladder, and mediates detrusor muscle relaxation.^33^ A beta-3 adrenoceptor agonist has recently been approved for treatment of overactive bladder symptoms.^34,35^ One conference abstract,^36^ and 2 published papers^37,38^ provided relevant information on the common rs4994 missense mutation, of which 2 could be included in metaanalysis. In the initial report, in a heterogeneous Japanese sample of 13 men and 31 women, with diverse urological pathologies including neurogenic bladder and benign prostatic hyperplasia, the rs4994 SNP was not associated with LUTS (odds ratio [OR], 1.20; 95% confidence interval [CI], 0.32–4.47).^36^ Results were not available stratified by sex, and could not be included in quantitative synthesis. Subsequent reports used larger samples of Japanese women,^37^ and Brazilian women^38^ (Table 1), and looked specifically at the overactive bladder phenotype, finding a large effect size (pooled OR, 2.46; 95% CI, 1.67–3.60) (Figure 2), with no heterogeneity. Despite a lack of information about genotyping quality control (QC), and some risk of population stratification, this large effect size confers some protection from bias, providing Venice grading BBB, or moderate epidemiological credibility (Table 2).

### *COL1A1*

rs1800012 also known as the Sp1-binding site polymorphism of collagen, type I, alpha 1, modifies transcription factor binding and gene expression. It has been most extensively studied in association with osteoporosis, where the minor allele is modestly associated with reduced bone mineral density and increased fracture risk.^39^ Collagen, type I, alpha 1 is a major structural component of the vaginal epithelium and endopelvic fascia. The available data on gene and protein expression in pelvic tissue from women with prolapse or stress incontinence are heterogeneous but suggest increased *COL1A1* expression with reduced type 1 collagen content.^40^ Seven studies provided data on the rs1800012 SNP in association with either POP or stress incontinence, of which 6 could be included in quantitative syntheses.

**Figure 3:**
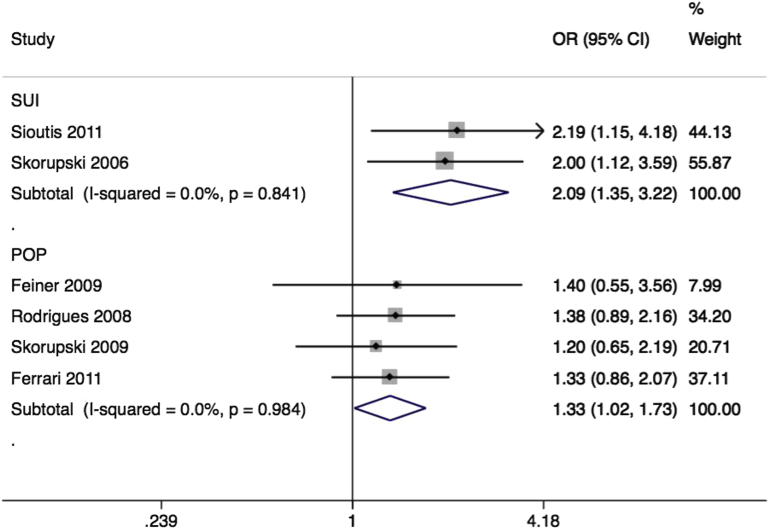
Forest plot of studies of rs1800013 SNP of COL1A1 Forest plot of studies^41-44,46-47^ reporting associations between rs1800012 single-nucleotide polymorphism (SNP)* of collagen type 1 alpha 1 gene and either stress urinary incontinence (SUI) or pelvic organ prolapse (POP). *RefSNP alleles G/T. Plot presented as risk associated with minor allele T. *CI*, confidence interval; *OR*, odds ratio. *Cartwright. Genetic association studies of LUTS and POP. Am J Obstet Gynecol 2015*.

Five studies reported associations of rs1800012 with anatomical POP in Brazilian,^41^ Israeli,^42^ Polish,^43^ Italian,^44^ and Korean^45^ populations (Table 1). The Korean study found only the wild type GG allele among all 30 participants, and could not be included in quantitative synthesis. Despite each individual study being underpowered, the pooled effect size for the remaining 4 studies was significant (OR, 1.33; 95% CI, 1.02–1.73) (Figure 3) with low inconsistency. With limited information about genotyping QC, and a possible risk of population stratification in 2 samples,^41,42^ we considered that bias could not be fully excluded, providing Venice grading BBB, or moderate epidemiological credibility (Table 2).

Two studies of Polish^46^ and Greek^47^ women reported associations of the same polymorphism with stress incontinence, in both cases using a combined symptomatic and objectively measured case definition. The pooled effect size was large (OR, 2.09; 95% CI, 1.35–3.22) (Figure 3) with no heterogeneity (I^2^ = 0%). There was significant deviation from Hardy-Weinberg equilibrium in one sample,^46^ suggesting significant potential for bias. However, exclusion of this study would not change the result. With high risk of bias the Venice grading was CBC, or weak epidemiological credibility (Table 2).

### *COL3A1*

**Figure 4:**
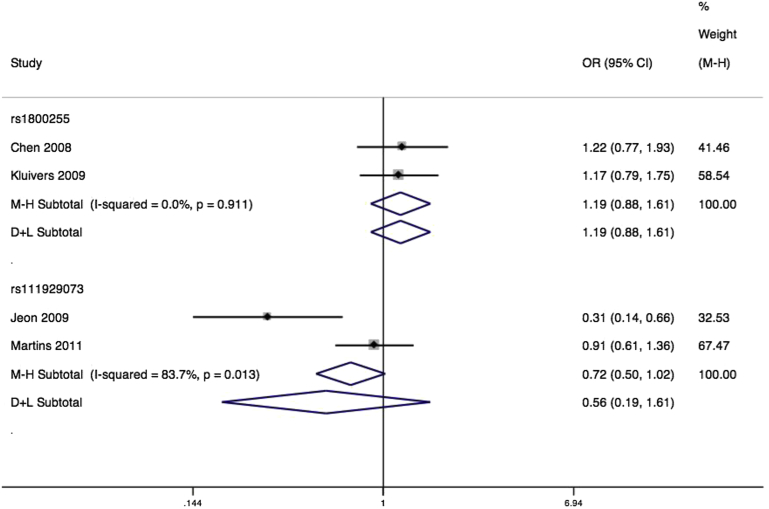
Forest plot of COL3A1 SNPs and prolapse Forest plot of studies^49-52^ reporting associations between rs1800255* and rs111929073* single-nucleotide polymorphisms (SNPs) of collagen type 3, alpha 1 gene and pelvic organ prolapse with either fixed or random effects models**. *For both SNPs RefSNP alleles A/G. Plot presented as risk associated with minor allele A. **Mantel-Haenszel fixed effects model (M-H)/DerSimonian and Laird random effects model (D+L). *CI*, confidence interval; *OR*, odds ratio. *Cartwright. Genetic association studies of LUTS and POP. Am J Obstet Gynecol 2015*.

A large number of mutations in collagen, type 3, alpha 1 have been associated with vascular Ehlers-Danlos syndrome. Inconsistent evidence suggests that urinary incontinence and prolapse may be prevalent among women with Ehlers-Danlos.^48^ Collagen, type 3 has a particular function in tissue repair, and is typically overexpressed in pelvic tissues from women with prolapse.^40^ We identified studies testing associations with 2 missense variants rs1800255 and rs111929073, as well as 1 synonymous SNP rs1801184. Both missense variants had been tested in 2 studies, and therefore could be combined in quantitative syntheses. Separate Taiwanese^49^ and Dutch^50^ studies found a nonsignificant pooled association between rs1800255 and anatomic prolapse (OR, 1.19; 95% CI, 0.88–1.61) (Figure 4), with no heterogeneity (Table 2).

For rs111929073, separate Korean^51^ and Brazilian^52^ samples demonstrated a nonsignificant pooled effect (OR, 0.56; 95% CI, 0.19–1.61) (Figure 4) with high heterogeneity (I^2^ = 83.7%, *P* < .01). Case definitions were similar for the 2 studies, making this an unlikely source of heterogeneity. The primary Korean study had suggested a large protective effect of the minor allele, and the heterogeneity between studies might instead be explained by differences in populations, or a simple Proteus effect.

### *LAMC1*

Laminin gamma 1 is 1 of 3 kinds of laminin chain that combine to make different laminin isoforms. These extracellular matrix glycoproteins are an important constituent of basement membranes, with roles in cell adhesion and migration. *LAMC1* was initially proposed as a candidate gene for prolapse in a linkage study of 9 individuals from a family affected by early-onset severe prolapse.^53^ We identified 3 further studies all from the United States that attempted to replicate this initial report of an association with rs10911193,^54-56^ with all 3 including testing of additional SNPs (Table 1).

**Figure 5:**
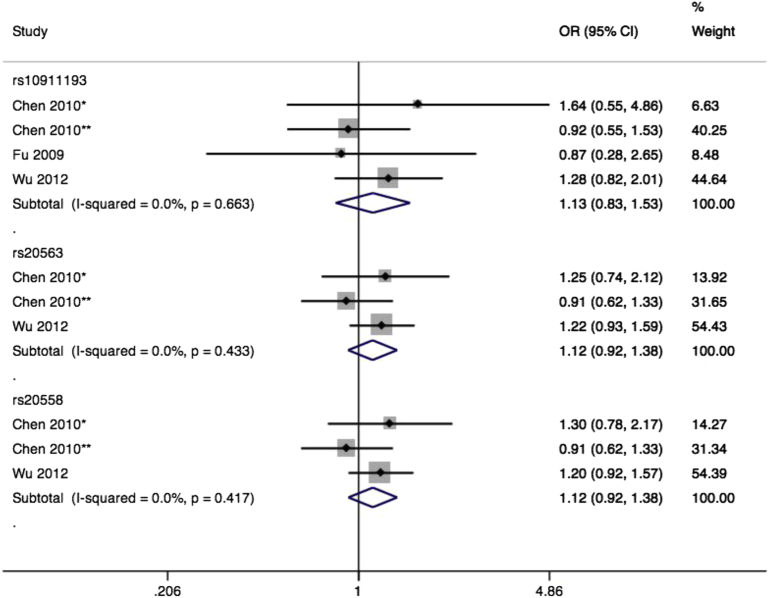
Forest plot of LAMC1 SNPs and prolapse Forest plot of studies^54-56^ reporting associations among rs10911193, rs20563, and rs20558 single-nucleotide polymorphisms (SNPs) of laminin gamma 1 gene and pelvic organ prolapse. *African American subsample. **White subsample. rs10911193 RefSNP alleles C/T. Plot presented as risk associated with minor allele T. rs20563 RefSNP alleles A/G. Plot presented as risk associated with minor allele A. rs20558 RefSNP alleles C/T. Plot presented as risk associated with minor allele C. *CI*, confidence interval; *OR*, odds ratio. *Cartwright. Genetic association studies of LUTS and POP. Am J Obstet Gynecol 2015*.

All 3 individual studies found no association for rs10911193, with a nonsignificant pooled effect (OR, 1.13; 95% CI, 0.83–1.53) (Figure 5) and no heterogeneity. There was no evidence of small study bias or publication bias. Genotyping QC was generally well documented for these studies, and population stratification appropriately accounted for. Two of the studies^54,55^ provided further data on rs20563 and rs20558, 2 missense SNPs in near perfect linkage disequilibrium, but again with nonsignificant pooled effects (both OR, 1.12; 95% CI, 0.92–1.38) (Figure 5) and no heterogeneity.

### *MMP1*

**Figure 6:**
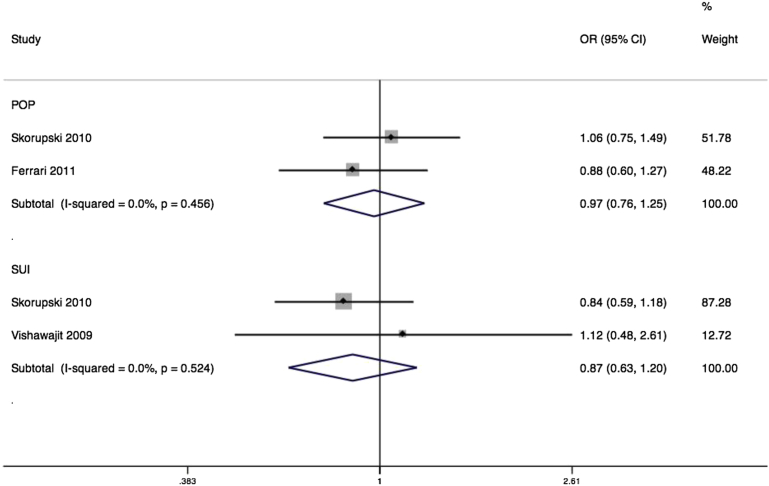
Forest plot of rs1799750 SNP of MMP1 Forest plot of studies^44,60,61^ reporting associations between rs1799750* single-nucleotide polymorphism (SNP) of matrix metalloproteinase 1 (MMP1) gene and either stress urinary incontinence (SUI) or pelvic organ prolapse (POP) with either fixed or random effects models. ∗RefSNP Alleles -/G. Plot presented as risk associated with minor deletion allele. *CI*, confidence interval; *OR*, odds ratio. *Cartwright. Genetic association studies of LUTS and POP. Am J Obstet Gynecol 2015*.

Matrix metalloproteinase-1, also known as interstitial collagenase, is one of a number of enzymes that cleave collagen type 1. The *MMP1* gene is up-regulated in pelvic tissues of women with prolapse.^40^ Common variants of this gene have been extensively studied in association with chronic obstructive pulmonary disease,^57^ cardiovascular disease,^58^ and a number of cancers including of lung, colon, and breast. We identified 2 unpublished studies from the United States,^59,60^ and 2 published studies of Polish^61,62^ and Italian^44^ samples assessing associations between MMP1 variants and stress incontinence or prolapse. Of these, 2 studies reported on rs1799750 in association with prolapse,^44,61^ with a nonsignificant pooled effect (OR, 0.97; 95% CI, 0.76–1.25) (Figure 6) with no heterogeneity. One of the 2 studies included demonstrated marked deviation from Hardy-Weinberg equilibrium, and exclusion of this study would however leave a single eligible study with a nonsignificant association (OR, 0.88; 95% CI, 0.60–1.27).^44^ For the 2 studies testing associations with SUI,^60,61^ the pooled effect was again nonsignificant (OR, 0.87; 95% CI, 0.63–1.20), with no heterogeneity.

### *MMP3*

**Figure 7:**
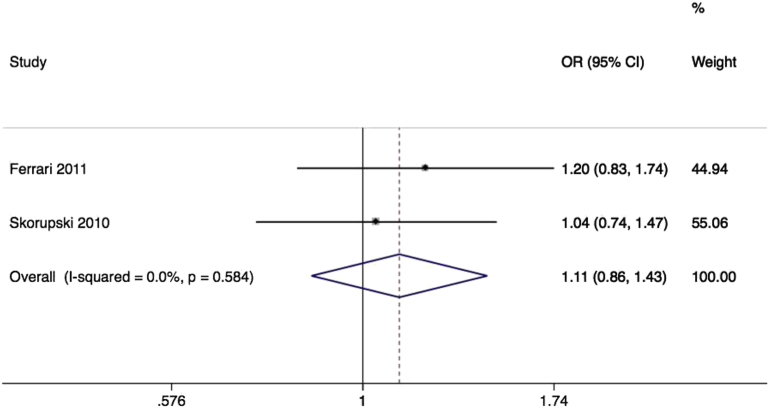
Forest plot of rs3025058 SNP of MMP3 and prolapse Forest plot of studies^6,44^ reporting associations between rs3025058* single-nucleotide polymorphism (SNP) of matrix metalloproteinase 3 gene and pelvic organ prolapse. *RefSNP Alleles -/T. Plot presented as risk associated with minor deletion allele. *CI*, confidence interval; *OR*, odds ratio. *Cartwright. Genetic association studies of LUTS and POP. Am J Obstet Gynecol 2015*.

Matrix metalloproteinase-3, also known as stromelysin-1, is an enzyme that degrades a number of extracellular matrix components including collagen type 3 and elastin. Similarly to *MMP1*, its common variants have received most research attention in association with cardiovascular disease,^58^ and a number of cancers. We identified 2 studies again of women of European descent,^44,61,62^ both testing associations of rs3025058, known as the 5A/6A promoter InDel, with prolapse. The pooled effect was again nonsignificant (OR, 1.11; 95% CI, 0.86–1.43) (Figure 7) with no heterogeneity.

### *MMP9*

**Figure 8:**
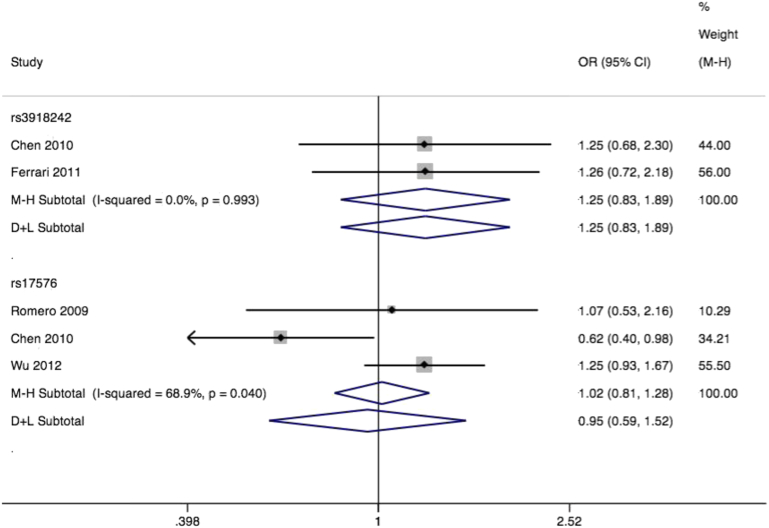
Forest plot of MM9 SNPs and prolapse Forest plot of studies^44,63-65^ reporting associations between rs3918242* and rs17576** single-nucleotide polymorphisms (SNPs) of matrix metalloproteinase 9 gene and pelvic organ prolapse with either fixed or random effects models⌘. *rs3918242 RefSNP alleles C/T. Plot presented as risk associated with minor allele T. **rs17576 RefSNP alleles A/G. Plot presented as risk associated with minor allele A. ⌘Mantel-Haenszel fixed effects model (M-H)/DerSimonian and Laird random effects model (D+L). *CI*, confidence interval; *OR*, odds ratio. *Cartwright. Genetic association studies of LUTS and POP. Am J Obstet Gynecol 2015*.

Matrix metalloproteinase-9, also known as 92-kDa type IV collagenase, degrades collagen type 4 and type 5. Some evidence suggests increased activation of MMP9 in pelvic tissues from women with prolapse.^63^ Like *MMP1* and *MMP3*, its common polymorphisms have been linked to chronic obstructive pulmonary disease,^57^ cardiovascular disease,^58^ and some cancers. We identified 4 studies of Italian,^44^ Taiwanese,^64^ and white US^63,65^ samples, assessing 10 different polymorphisms in association with prolapse. Three studies contributed to a metaanalysis of the rs17576 missense polymorphism. The pooled effect was nonsignificant (OR, 1.02; 95% CI, 0.81–1.28) (Figure 8) but with significant heterogeneity (I^2^ = 68.9%, *P* = .04). Case definitions were similar for the 3 studies, making this an unlikely source of heterogeneity. All studies demonstrated Hardy-Weinberg equilibrium, and we judged a low risk of population stratification. The single study among Asian women^64^ suggested a narrowly significant effect (OR, 0.62; 95% CI, 0.40–0.98), while subgroup analysis of the 2 white US samples showed no pooled effect (OR, 1.22; 95% CI, 0.93–1.60). Two studies contributed to metaanalysis of rs3918242, with a nonsignificant effect (OR, 1.25; 95% CI, 0.83–1.89) (Figure 8) and no heterogeneity.

### Publication bias and selective analysis

Each metaanalysis included at most 4 studies or subgroups, providing low power for conventional measures of funnel plot asymmetry. The Harbord test demonstrated no evidence of small study bias or publication bias (all *P* > .1). We applied the significance chasing bias test,^30^ to look for further evidence of publication bias or selective outcome reporting. This exploratory test is used to detect an excess of significant results, either within a single metaanalysis, or in a whole domain of research. In common with other tests of publication bias, *P* < .1 is usually taken as the threshold for significance. We applied the test across each of the 13 metaanalyses conducted individually, and for the 13 considered together as 1 domain. Given the power of the individual studies to detect the observed pooled effect sizes in each metaanalysis, across the domain as a whole we expected 6.61 statistically significant studies, and observed 7 significant studies in our own prespecified reanalyses using the allelic test (*P* = .87). However, primary publications applied a variety of analytic techniques, and from the set of studies included in metaanalysis we observed 11 studies reporting statistically significant results in their own analyses (*P* = .14), typically using alternative models of inheritance. These findings are suggestive primarily of selective analysis, rather than publication bias. Individual metaanalyses again provided limited power for this test, but possible bias was most apparent in the quantitative synthesis of association of prolapse with the rs17576 SNP of MMP9 (*P* = .11).

### Genes and/or polymorphisms reported in a single study

Among the included studies, some had assessed associations with polymorphisms for which no replication has been reported. Statistically significant associations have been suggested between prolapse and the rs2228480 polymorphism of *ESR1*, the estrogen receptor alpha^66^; between prolapse and certain haplotypes of *ESR2*, the estrogen receptor beta^67^; between prolapse and the rs484389 polymorphism of *PGR*, the progesterone receptor^68^; between prolapse and the rs10478694 polymorphism of *EDN1*, endothelin 1^69^; between incontinence and the CAG copy number variant of *AR*, the androgen receptor^70^; between incontinence and the rs6313 polymorphism of *HTR2A*, the serotonin 2A receptor^71^; between stress incontinence and both the rs2165241 and rs1048661 variants of *LOX-L1*, lysyloxidaselike-1^72^; between the rs1136410 polymorphism of poly-ADP ribose polymerase (*PARP*)^73^ and prolapse; and finally between the rs1695 polymorphism of glutathione S-transferase pi (*GSTP1*) and prolapse.^74^ We found only 1 published study reporting entirely nonsignificant results,^75^ further suggesting a high probability of selective outcome reporting or publication bias for this field of study as a whole. Following the Venice recommendations,^28^ we a priori assigned all nominally significant but unreplicated associations weak epidemiological credibility. Three genome-wide association studies (GWAS) have now been reported for incontinence or prolapse.^76-78^ Of note, none of these suggested candidates for prolapse or incontinence, including both those from single studies, as well as those included in metaanalyses, were identified in these genome-wide analyses. Across the 3 GWAS, SNPs at 9 independent loci have reached genome-wide significance (*P* < 5 × 10^-8^) (Table 1) in discovery cohorts, although replication of these candidate loci has not been demonstrated.

## Comment

### Strengths and limitations

The strengths of this review include a comprehensive search of both published and unpublished studies, applying explicit criteria to potentially eligible studies, and employing standardized, piloted data forms for data collection, guided by written instructions, and an unbiased assessment and synthesis of reported associations. We followed a prespecified data analysis plan, and contacted authors for clarifications and additional data.

Among the challenges faced in this review was the inclusion of studies with varying diagnostic criteria. There may be considerable disparity between symptomatic and objective findings for both LUTS and prolapse, and despite long-standing efforts for standardization^2^ diagnostic criteria are not widely agreed upon. Despite this caution, we found that the literature had used largely concordant definitions. From the prolapse studies, 2 studies had used a prolapse case definition based on need for surgical treatment, but all others used an accepted anatomic staging system, typically POP Quantification. There was also little variation in cutoffs for significant prolapse, with almost all studies considering prolapse stage 0 or stage 1 as normal/control. Both overactive bladder studies included in metaanalysis used a combination of self-reported symptoms, with 3-day bladder diary for diagnosis. Similarly both SUI studies included in metaanalysis used a combination of cystometry and pad testing for diagnosis. This is reflected in a lack of heterogeneity in most metaanalyses. Regardless of the presence of statistical heterogeneity, there remains potential for bias toward the null from heterogeneity in case definitions.

It is evident that overactive bladder in particular may have multiple underlying causes,^79,80^ and these syntheses may therefore include participants with diverse underlying etiologies for their symptoms. The largest metaanalyses possible still include <1000 participants in total, and therefore provide adequate power only for associations with large effect size (approximately OR, ≤0.6 or OR, ≥1.8). It is both likely that smaller effect sizes have been missed in these syntheses, and highly probable that polymorphisms with larger effect sizes are still to be discovered.

### Future work

Future advances are likely within the context of GWAS using large-scale population-based cohorts phenotyped for these conditions. The discovery of further causative variants should both help to explain the complex pathophysiology of these conditions, and provide potentially a route to effective prevention and treatment.

### Conclusions

Family and twin studies have provided convincing evidence for genetic predisposition to incontinence, prolapse, and overactive bladder, with genetic variation contributing up to half of population phenotypic variability. These metaanalyses provide moderate epidemiological credibility for associations of variation in *ADRB3* with overactive bladder, and *COL1A1* with prolapse. As for all complex diseases, these 2 currently identified polymorphisms explain a tiny fraction of that phenotypic variation. The widespread availability of direct-to-consumer testing means that some patients may present with questions about the implications of these polymorphisms. However, testing for any of these SNPs cannot be recommended based on current evidence. Nevertheless, clinicians and researchers should be aware of the putative risks associated with these SNPs, and the uncertainty regarding potential biases in the primary studies. In the future, genetic counseling may play one part of advice about risks of mode of delivery, and may help target women for primary or secondary prevention. Currently, clinicians should continue to use a family history of prolapse or incontinence as a simple marker of future risk, with clearly documented interactions with modifiable risk factors such as vaginal childbirth and obesity.

## Figures and Tables

**Supplementary Figure:**
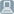
Summary of interim Venice guideline ratings of credibility of genetic associations Strong credibility for association requires AAA rating. Any B rating confers maximum moderate credibility, while any C rating confers weak credibility. *Cartwright. Genetic association studies of LUTS and POP. Am J Obstet Gynecol 2015*.

Adapted and abridged from Ioannidis et al.^29^

**Supplementary Table 1:** Functional annotation of investigated genes

Official gene symbol	Gene name(s)	No. of studies included	Molecular function(s)
*ADRA1A*	Adrenergic, alpha-1A-, receptor	1	Adrenoceptor activity Alpha-adrenergic receptor activity Alpha1-adrenergic receptor activity Amine receptor activity
*ADRB3*	Adrenergic, beta-3-, receptor	3	Adrenoceptor activity Beta-adrenergic receptor activity Amine receptor activity Beta-3-adrenergic receptor activity Adrenergic receptor binding Beta-3 adrenergic receptor binding Identical protein binding Protein homodimerization activity Amine binding Alcohol binding Protein dimerization activity Epinephrine binding Norepinephrine binding
*AR*	Androgen receptor	1	DNA binding Transcription factor activity Steroid hormone receptor activity Ligand-dependent nuclear receptor activity Androgen receptor activity Steroid binding Androgen binding Zinc ion binding Lipid binding Transcription activator activity Transcription regulator activity Hormone binding Ion binding Cation binding Sequence-specific DNA binding Metal ion binding Transition metal ion binding Protein dimerization activity
*COL1A1*	Collagen, type I, alpha 1	7	Structural molecule activity Extracellular matrix structural constituent Growth factor binding Identical protein binding Platelet-derived growth factor binding,
*COL3A1*	Collagen, type III, alpha 1	4	Integrin binding Structural molecule activity Extracellular matrix structural constituent Growth factor binding Protein complex binding SMAD binding Platelet-derived growth factor binding
*COL18A1*	Collagen, type XVIII, alpha 1	1	Structural molecule activity Extracellular matrix structural constituent Zinc ion binding Ion binding Cation binding Metal ion binding Transition metal ion binding
*CPE*	Carboxypeptidase E	1	Metallocarboxypeptidase activity Zinc ion binding
*CYP17A1*	Cytochrome P450, family 17, subfamily A, polypeptide 1	1	Steroid 17-alpha-monooxygenase activity Iron ion binding Steroid hydroxylase activity Electron carrier activity Oxygen binding Heme binding Ion binding Cation binding Metal ion binding Tetrapyrrole binding Transition metal ion binding
*CYP19A1*	Cytochrome P450, family 19, subfamily A, polypeptide 1	1	Iron ion binding Electron carrier activity Oxidoreductase activity Oxygen binding Heme binding Ion binding Cation binding Metal ion binding Tetrapyrrole binding Transition metal ion binding Aromatase activity
*EDN1*	Endothelin 1	1	Hormone activity Bombesin receptor binding Endothelin A receptor binding Endothelin B receptor binding
*ESR1*	Estrogen receptor 1	1	DNA binding Transcription factor activity Steroid hormone receptor activity Ligand-dependent nuclear receptor activity Steroid binding Zinc ion binding Lipid binding Promoter binding Nitric-oxide synthase regulator activity Estrogen receptor activity Transcription regulator activity Estrogen response element binding Hormone binding Ion binding Cation binding Sequence-specific DNA binding Metal ion binding transition Metal ion binding Protein N-terminus binding
*ESR2*	Estrogen receptor 2 (ER beta)	1	DNA binding Transcription factor activity Steroid hormone receptor activity Transcription cofactor activity Transcription coactivator activity Ligand-dependent nuclear receptor activity Steroid binding transcription factor binding Zinc ion binding Lipid binding Transcription activator activity Estrogen receptor activity Transcription regulator activity Receptor regulator activity Receptor inhibitor activity Hormone binding Ion binding Cation binding Sequence-specific DNA binding Metal ion binding Transition metal ion binding Receptor antagonist activity
*GSTM1*	Glutathione S-transferase mu 1	1	Glutathione transferase activity
*GSTP1*	Glutathione S-transferase pi 1	1	Glutathione transferase activity
*GSTT1*	Glutathione S-transferase theta 1	1	Glutathione transferase activity
*HTR2A*	5-Hydroxytryptamine (serotonin) receptor 2A	1	Serotonin receptor activity Amine receptor activity Protein complex binding Amine binding Serotonin binding
*LAMC1*	Laminin, gamma 1 (formerly LAMB2)	3	Structural molecule activity Extracellular matrix structural constituent,
*LOXL1*	Lysyl oxidase-like 1	3	Copper ion binding Oxidoreductase activity Ion binding Cation binding Metal ion binding Transition metal ion binding
*MMP1*	Matrix metallopeptidase 1 (interstitial collagenase)	5	Endopeptidase activity Metalloendopeptidase activity Calcium ion binding Peptidase activity Metallopeptidase activity Zinc ion binding Ion binding Cation binding Metal ion binding Transition metal ion binding Peptidase activity Acting on L-amino acid peptides
*MMP10*	Matrix metallopeptidase 10 (stromelysin 2)	1	Endopeptidase activity Metalloendopeptidase activity Calcium ion binding Peptidase activity Metallopeptidase activity Zinc ion binding Ion binding Cation binding Metal ion binding Transition metal ion binding Peptidase activity Acting on L-amino acid peptides
*MMP11*	Matrix metallopeptidase 11 (stromelysin 3)	1	Endopeptidase activity Metalloendopeptidase activity Calcium ion binding Peptidase activity Metallopeptidase activity Zinc ion binding Ion binding Cation binding Metal ion binding Transition metal ion binding Peptidase activity Acting on L-amino acid peptides
*MMP2*	Matrix metallopeptidase 2 (gelatinase A, 72-kDa gelatinase, 72-kDa type IV collagenase)	1	Endopeptidase activity Metalloendopeptidase activity Calcium ion binding Peptidase activity Metallopeptidase activity Zinc ion binding Ion binding Cation binding Metal ion binding Transition metal ion binding Peptidase activity Acting on L-amino acid peptides
*MMP3*	Matrix metallopeptidase 3 (stromelysin 1, progelatinase)	3	Endopeptidase activity Metalloendopeptidase activity Calcium ion binding Peptidase activity Metallopeptidase activity Zinc ion binding Ion binding Cation binding Metal ion binding Transition metal ion binding Peptidase activity Acting on L-amino acid peptides
*MMP8*	Matrix metallopeptidase 8 (neutrophil collagenase)	1	Endopeptidase activity Metalloendopeptidase activity Calcium ion binding Peptidase activity Metallopeptidase activity Zinc ion binding Ion binding Cation binding Metal ion binding Transition metal ion binding Peptidase activity Acting on L-amino acid peptides
*MMP9*	Matrix metallopeptidase 9 (gelatinase B, 92-kDa gelatinase, 92-kDa type IV collagenase)	4	Endopeptidase activity Metalloendopeptidase activity Calcium ion binding Peptidase activity Metallopeptidase activity Zinc ion binding Ion binding Cation binding Metal ion binding Transition metal ion binding Peptidase activity Acting on L-amino acid peptides
*PARP1*	Poly (ADP-ribose) polymerase 1	1	Telomere maintenance DNA repair Transcription Chromosome organization
*PGR*	Progesterone receptor	1	DNA binding transcription factor activity Steroid hormone receptor activity Ligand-dependent nuclear receptor activity Steroid binding Zinc ion binding Lipid binding Transcription regulator activity Ion binding Cation binding Sequence-specific DNA binding Metal ion binding Transition metal ion binding Protein N-terminus binding
*PRCP*	Prolylcarboxypeptidase (angiotensinase C)	1	Carboxypeptidase activity Serine hydrolase activity
*TIMP1*	TIMP metallopeptidase inhibitor 1	1	Enzyme inhibitor activity, Endopeptidase inhibitor activity Metalloendopeptidase inhibitor activity Metalloenzyme regulator activity Peptidase inhibitor activity Metalloenzyme inhibitor activity
*TIMP3*	TIMP metallopeptidase inhibitor 3	1	Enzyme inhibitor activity Endopeptidase inhibitor activity Metalloendopeptidase inhibitor activity Metalloenzyme regulator activity Peptidase inhibitor activity Metalloenzyme inhibitor activity
*ZFAT*	Zinc finger and AT hook domain containing	1	DNA binding Zinc ion binding Ion binding Cation binding Metal ion binding Transition metal ion binding

Functions assigned from http://david.abcc.ncifcrf.gov. *Cartwright. Genetic association studies of LUTS and POP. Am J Obstet Gynecol 2015*.

**Supplementary Table 2:** Overrepresented Gene Ontology biological processes among investigated genes

Biological process	GO term	Description	Genes
1	GO:0030574	Collagen catabolic process	*MMP1 MMP2 MMP3 MMP8 MMP9 MMP10 MMP11*
2	GO:0005578	Proteinaceous extracellular matrix	*LOXL1 MMP1 MMP2 MMP3 MMP8 MMP9 MMP10 MMP11 TIMP1*
3	GO:0006508	Proteolysis	*CPE MMP1 MMP2 MMP3 MMP8 MMP9 MMP10 MMP11 PRCP*
4	GO:0008152	Metabolic process	*CPE GSTM1 MMP2 MMP8 MMP3 GSTP1 MMP1 MMP10 MMP9*
5	GO:0005576	Extracellular region	*COL1A1 COL3A1 CPE EDN1 ESR2 LAMC1 LOXL1 MMP1 MMP2 MMP3 MMP8 MMP9 MMP10 MMP11 TIMP1 COL18A1*
6	GO:0007267	Cell-cell signalling	*PGR AR ADRA1A ESR2 EDN1*
7	GO:0005615	Extracellular space	*AR COL1A1 COL3A1 EDN1 LAMC1 LOXL1 MMP2 MMP3 MMP8 MMP9 MMP10 COL18A1*
8	GO:0008270	Zinc ion binding	*ESR1 ESR2 MMP1 MMP2 MMP3 MMP8 MMP9 MMP10 MMP11 PGR ZFAT*

All processes significant at *P* < .01, analysis using http://webclu.bio.wzw.tum.de/profcom/. Overrepresented Gene Ontology: http://www.geneontology.org. *Cartwright. Genetic association studies of LUTS and POP. Am J Obstet Gynecol 2015*.

**Table 1:** Included studies

Study	Journal and year	Country	Descent, ethnicity, race^a^	Gene symbols(s)	Polymorphism(s) dbSNP ID	Case definition	Control definition	Cases genotyped, n	Controls genotyped, n
Allen-Brady et al^76^	Obstet Gynecol 2011	United States, The Netherlands	White and Northern European descent	*LINC0108*^*b*^ *ZFAT* Intergenic Intergenic Intergenic *COL18A1*	rs1455311 rs1036819 rs430794 rs8027714 rs1810636 rs2236479	Surgically treated/recurrent POP with family history	Population controls	191	3036
Campeau et al^59^	Neurourol Urodyn 2011 (ICS abstract)	United States	Not stated	*MMP1*	rs1144393 rs498186 rs473509	Surgically treated POP	Hospital controls “without POP”	63	93
Chen et al^55^	Am J Obstet Gynecol 2010	United States	African American and Caucasian	*LAMC1*	rs10911193 rs20563 rs20558	POP stage >II	POP stage <II	165	246
Chen et al^66^	Int Urogynecol J 2008	Taiwan	Taiwanese	*ESR1*	rs17847075 rs2207647 rs2234693 rs3798577 rs2228480	POPQ ≥2	POPQ <2	88	153
Chen et al^68^	Acta Obstet Gynecol 2009	Taiwan	Taiwanese	PGR	rs500760 rs484389	POPQ ≥2	POPQ <2	87	150
Chen et al^78^	Am Soc Hum Genet 2013	United States	African American and Hispanic American	*PRCP*^*b*^	rs2086297	Symptomatic SUI	No SUI	≈3343	≈8183
Chen et al^66^	Int Urogynecol J 2008	Taiwan	Taiwanese	*COL3A1*	rs1800255 rs1801184	POPQ ≥2	POPQ <2	84	147
Chen et al^64^	Eur J Obstet Gynecol 2010	Taiwan	Taiwanese	*MMP9*	rs3918242 rs17576 rs2250889	POPQ ≥2	POPQ <2	92	152
Chen et al^67^	Eur J Obstet Gynecol 2008	Taiwan	Taiwanese	*ESR2*	rs2987983 rs1271572 rs944459 rs1256049 rs1255998	POPQ ≥2	POPQ <2	69	141
Cho et al^45^	Yonsei Med J 2009	Korea	Korean	*COL1A1*	rs1800012	Surgically treated POPQ ≥3	POPQ = 0	15	15
Choy et al^69^	ICS abstract 2007	Hong Kong	Chinese	*EDN1*	rs5370 rs10478694	POPQ ≥2	Hospital “normal’’ controls and HapMap Han Chinese controls	60 (rs5370) and 67 (rs10478694)	210
Cornu et al^70^	World J Urol 2011	France	Caucasian	*ESR1* *CYP17A1* *CYP19A1* *AR*	rs2234693 rs743572 rs60271534 CAG repeat	Treated for UI (30 UUI, 107 SUI)	No UI or OAB	121	66
Feiner et al^42^	Int Urogynecol J 2009	Israel	Caucasian or Ashkenazi-Jewish	*COL1a1*	rs1800012	POPQ ≥3	POPQ <2	36	36
Ferrari et al^44^	Arch Gynecol Obstet 2012	Italy	Italian	*COL1a1* *MMP9* *MMP1* *MMP3*	rs1800012 rs3918242 rs1799750 rs3025058	POPQ ≥2	POPQ <2	137	96
Ferreira et al^38^	Am J Obstet Gynecol 2011	Brazil	White or nonwhite	*ADRB3*	rs4994	Symptomatic OAB without severe SUI	No LUTS	49	169
Ferrell et al^75^	Reprod Sci 2009	United States	African American or Caucasian	*LOXL1*	rs16958477	POP stage ≥II	POP stage <II	137	130
Fu et al^56^	J Urol 2009 (AUA abstract)	United States	Not stated	*LAMC1* *LOXL1*	rs10911193	POP stage ≥III	No POP or UI	61	33
Honda et al^37^	Neurourol Urodyn 2014	Japan	Japanese	*ADRb3*	rs4994	Symptomatic OAB	No OAB	100	101
Jeon et al^51^	J Urol 2009	Korea	Korean	*COL3a1*	rs111929073	POPQ ≥2	POPQ <2 and no SUI	36	36
Kim et al^74^	Eur J Obstet Gynecol Reprod Biol 2014	Korea	Korean	*GSTM1* *GSTT1* *GSTP1*	Null Null rs1695	POPQ ≥3	POPQ <2	189	156
Kim et al^73^	Menopause 2014	Korea	Korean	*PARP1*	rs1136410	POPQ ≥3	POPQ <2	185	155
Lince et al^50^	Int Urogynecol J 2014	The Netherlands	≈99% Dutch	*COL3a1*	rs1800255	POPQ ≥2	POPQ <2	272	82
Martins et al^52^	Neurourol Urodyn 2011	Brazil	White or nonwhite	*COL3a1*	rs111929073	POP stage ≥III	POP stage <II	107	209
Noronha et al^71^	J Investig Med 2010	Brazil	Predominant European/white	*HTR2A*	rs6313	Symptomatic UI	Self-reported continent women, and population controls	68	849
Ozbek et al^72^	J Obstet Gynaecol Res 2013	Turkey	Caucasian	*LOXL1*	rs2165241 rs3825942 rs1048661	Symptomatic SUI	No UI	93	75
Rodrigues et al^41^	Int Urogynecol J 2008	Brazil	White or nonwhite	*COL1a1*	rs1800012	POP stage ≥III	POP stage <II and no SUI	107	209
Romero and Jamison^65^	J Pelv Med Surg 2008	United States	White	*MMP1* *MMP2* *MMP3* *MMP8* *MMP9* *MMP10* *MMP11* *TIMP1* *TIMP3*	rs2071230 rs7201 rs679620 rs35866072 rs17576 rs17435959 rs738789 rs4898 rs2016293	POPQ ≥3	POPQ <2 and no UI	45	38
Sioutis et al^47^	Int Urogynecol J 2011	Greece	Greek	*COL1a1*	rs1800012	SUI confirmed with urodynamics and positive pad test, and postmenopausal	Healthy postmenopausal	45	45
Skorupski^43^	Int Urogynecol J 2009 (IUGA abstract)	Poland	Polish	*COL1a1*	rs1800012	POPQ ≥2	POPQ <2 and no UI	120	97
Skorupski et al^46^	Am J Obstet Gynecol 2006	Poland	Polish	*COL1a1*	rs1800012	SUI confirmed with urodynamics and positive pad test	POPQ <2 and no UI	50	50
Skorupski et al^61^	Ginekol Polska 2010	Poland	Polish	*MMP1* *MMP3*	rs1799750 rs3025058	POPQ ≥2	POPQ <2	132	133
Takeda et al^36^	ICS Abstract 2002	Japan	Japanese	*ADRb3* *ADRA1A*	rs4994 rs1048101	Any LUTS (includes mixed group of women and men)	No LUTS	27	17
Velez Edwards et al^77^	Am Soc Hum Gen 2013	United States	African American and Hispanic American	*CPE*^b^ Intergenic	rs28573326 rs113518633	POP stage ≥I	POP stage = 0	1427	1274
Vishwajit et al^60^	ICS abstract 2009	United States	Not stated	*MMP1*	rs1799750	SUI with varying POP	Neither SUI nor POP	40	15
Wu et al^54^	Am J Obstet Gynecol 2012	United States	Non-Hispanic white	*LAMC1*	rs10911193 rs1413390 rs20558 rs20563 rs10911206 rs2296291 rs12041030 rs12739316 rs3768617 rs2483675 rs10911211 rs41475048 rs1058177 rs12073936	POPQ ≥3	POPQ <2	239	197
Wu et al^63^	Obstet Gynecol 2012	United States	Non-Hispanic white	*MMP9*	rs3918253 rs3918256 rs3918278 rs17576 rs2274755 rs17577 rs2236416 rs3787268	POPQ ≥3	POPQ <2	239	197

*AUA*, American Urological Association; *ICS*, International Continence Society; *IUGA*, International Urogynecological Association; *LUTS*, lower urinary tract symptoms; *OAB*, overactive bladder; *POP*, pelvic organ prolapse; *POPQ*, Pelvic Organ Prolapse Quantification system; *SNP*, single-nucleotide polymorphism; *SUI*, stress urinary incontinence; *UI*, urinary incontinence; *UUI*, urge urinary incontinence. *Cartwright. Genetic association studies of LUTS and POP. Am J Obstet Gynecol 2015*.

a Assessments of descent/ethnicity/race as specified in primary publications, or from additional data from authors, or assumed for countries with low ethnic heterogeneity including Taiwan, Korea, and Japan

b Genome-wide significant genes (*P* <5 × 10^-8^) reported in genome-wide association study.

**Table 2:** Interim Venice assessments of epidemiological credibility for each metaanalysis

Gene	SNP	Phenotype	Studies, n	Sample with minor allele^a^	Pooled OR	I^2^ %	Deviation from HWE^b^	Proteus effect	Harbord test *P* value	Funnel plot	Genotyping QC	Risk of population stratification	Venice rating	Overall credibility
*ADRB3*	rs4994	OAB	2	136	2.46	0.0	None	None	n/a	n/a	Not reported	Yes^38^^c^	BBB	Moderate
*COL1A1*	rs1800012	SUI	2	92	2.09	0.0	Yes^46^	None	n/a	n/a	Not reported	Low	CBC	Weak
		POP	4	249	1.33	0.0	None	None	.88	Symmetric	Not reported	Yes^41,42^^c^	BBB	Moderate
*COL3A1*	rs1800255	POP	2	257	1.19	0.0	None	Yes	n/a	n/a	Not reported^49^/appropriate^50^	None	BCB	Weak
	rs111929073	POP	2	115	0.56	83.7	None	None	n/a	n/a	Not reported	Yes^52^^c^	BCB	Weak
*LAMC1*	rs10911193	POP	4	218	1.12	0.0	None	None	.97	Symmetric	Appropriate^54,55^/not reported^56^	Low	BCB	Weak
	rs20563	POP	3	525	1.12	0.0	None	None	.86	Symmetric	Appropriate	Low	BCA	Weak
	rs20558	POP	3	551	1.12	0.0	None	None	.93	Symmetric	Appropriate	Low	BCA	Weak
*MMP1*	rs1799750	POP	2	234	0.83	74.9	Yes^61^	Yes	n/a	n/a	Not reported	Low	BCC	Weak
		SUI	2	150	0.88	3.4	None	None	n/a	n/a	Not reported	Yes^60^^c^	BCC	Weak
*MMP3*	rs3025058	POP	2	381	1.11	0.0	Yes^61^	None	n/a	n/a	Not reported	Low	BCC	Weak
*MMP9*	rs3918242	POP	2	99	1.25	0.0	None	None	n/a	n/a	Not reported	Low	CCC	Weak
	rs17576	POP	3	473	1.05	68.9	None	None	.72	Symmetric	Not reported^57^/appropriate^54^	Low	BCB	Weak

Three-letter code corresponds to A through C ratings of amount of evidence, its consistency, and its protection from bias (Supplementary Figure). *HWE*, Hardy Weinberg Equilibrium; *OAB*, overactive bladder; *OR*, odds ratio; *POP*, pelvic organ prolapse; *QC*, quality control; *SNP*, single-nucleotide polymorphism; *SUI*, stress urinary incontinence. *Cartwright. Genetic association studies of LUTS and POP. Am J Obstet Gynecol 2015*.

a Pooled sample size of participants with minor allele

b Checked in controls and whole population, and metaanalysis rechecked excluding studies with significant departure

c Studies each include populations with mixed descent groups without reported adjustment.
